# tRNA-Uridine Aminocarboxypropyltransferase DTW Domain Containing 2 Suppresses Colon Adenocarcinoma Progression

**DOI:** 10.1155/2023/4354536

**Published:** 2023-09-16

**Authors:** Yun Qian, Yu-Jiang Li, Yi-Wei Fu, Cui-Xia Liu, Juan Wang, Bin Yang

**Affiliations:** Department of Digestive, Taizhou People's Hospital Affiliated to Nanjing Medical University, Taizhou 225300, China

## Abstract

**Background:**

DTW Domain Containing 2 (DTWD2) is a newly identified transfer RNA-uridine aminocarboxypropyltransferase. Dysregulated expression of DTWD1 has been reported in several malignancies, nevertheless, the role of DTWD2 in cancers remains completely unknown. Here, we aimed to initially investigate the expression and role of DTWD2 in colon adenocarcinoma.

**Methods:**

We first evaluated the transcription and mRNA levels of DTWD2 using data from The Cancer Genome Atlas. Besides, we tested its mRNA and protein expression in our enrolled retrospective cohort. Univariate and multivariate analyses were conducted to assess its prognostic value. Cellular experiments and xenografts were also performed to validate the role of DTWD2 in colon cancer progression.

**Results:**

DTWD2 was downregulated in colon adenocarcinoma and associated with poor prognosis. Lymph node metastasis, distant metastasis, and advanced tumor stage are all characterized by lower DTWD2 levels. Furthermore, Cox regression analysis demonstrated that DTWD2 is a novel independent prognostic factor for colon cancer patients. Finally, cellular and xenograft data demonstrated that silencing DTWD2 significantly enhanced colon cancer growth.

**Conclusion:**

Low expression of DTWD2 may be a potential molecular marker for poor prognosis in colon cancer.

## 1. Introduction

Transfer RNA (tRNA) links a specific codon in mRNA with its corresponding amino acid during protein synthesis. Similar to proteins, tRNAs can be post-transcriptionally modified by various enzymes [1]. For example, the 3-(3-amino-3-carboxypropyl)uridine (acp3U) is a conserved modification in bacteria and eukaryotes. In humans, acp^3^U is identified at positions 20 and 20a in the D-loop of certain tRNAs [2]. As for the upstream regulators, DTW Domain Containing 1 (DTWD1) is responsible for acp^3^U_20_ modification, whereas DTWD2 was responsible for acp^3^U_20a_ [3]. Therefore, DTWD proteins were also named as tRNA-Uridine aminocarboxypropyltransferases.

It is well-acknowledged that the defection of tRNA modification is responsible for numerous diseases, such as neurological disorders and malignancies [4]. Therefore, dysregulated DTWDs are also involved in human diseases. For example, *DTWD1* is speculated as a candidate gene in the development of the bipolar disorder and depressive disorder [5, 6]. Ma et al. reported that DTWD1 was a tumor suppressor in gastric cancer, which functioned by suppressing cyclin B1 expression and modulated by histone deacetylase 3 [7]. Consistently, *DTWD1* mRNA was significantly downregulated in breast cancer compared with non-cancerous breast tissues, suggesting its diagnostic significance [8]. Another study also reported the correlation between DTWD1 expression and melanoma prognosis [9]. Moreover, transcriptome data revealed *DTWD1* as a critical gene for subgrouping clear cell renal cell carcinoma into different risk groups [10].

However, few studies reported the clinical relevance of DTWD2 since it has been a newly identified enzyme in the past decade. Copy number variation of *DTWD2* was reported in primary open-angle glaucoma [11]. The single nucleotide polymorphisms of *DTWD2* may also be involved in scrub typhus according to bioinformatics analysis [12]. Until now, there is no reported evidence suggesting the involvement of DTWD2 in any malignancy.

Colorectal cancer is recognized as one of the most prevalent malignancies globally [13]. Despite advances in curative surgical resection and adjuvant therapies, the prognosis for regional advanced colon adenocarcinoma, specifically TNM stage III colon adenocarcinoma (COAD), remains suboptimal. Hence, the identification of specific biomarkers is not only crucial for prognostic prediction but also imperative for innovative therapeutic strategies. In this study, we have presented, for the first time, the involvement of DTWD2 in COAD. We have conducted a comprehensive analysis of the clinical relevance of DTWD2 in both The Cancer Genome Atlas (TCGA) cohort and a retrospective COAD cohort from our hospital. Additionally, we have explored the detailed function of DTWD2 in COAD progression through meticulous cellular experiments and mice models. The discovery of novel prognostic biomarkers carries significant significance in the context of chemotherapy and the risk of therapeutic failure in COAD [14]. Chemotherapy, being a standard treatment for COAD, can face challenges, such as the development of resistance, leading to therapeutic failure, and disease progression [15]. Hence, the identification of reliable prognostic biomarkers, such as DTWD2, has the potential to play a pivotal role in predicting treatment outcomes and guiding personalized therapeutic strategies for COAD patients [16]. This highlights the urgent need for improved prognostic tools in the management of COAD.

## 2. Methods

### 2.1. Ethical Approval

Written informed consent was obtained from each participant. This study was approved by the Ethics Committee of Taizhou People's Hospital (No. KY 2022-169-01).

### 2.2. Patient Enrollment and Follow Up

We retrospectively enrolled adult COAD patients who underwent surgical intervention in our hospital. The inclusion criteria included adult patients with COAD who underwent surgical intervention at Taizhou People's Hospital, and resected specimens confirmed as TNM stage III based on pathological tests. The exclusion criteria were patients who did not underwent radical surgery, lacked intact follow-up information, combined with other malignancy disease history, accepted pre-operative chemotherapy or radiotherapy, and disagreed with the written informed consent. After exclusion, 176 cases were enrolled in the final cohort from our medical center (Taizhou, China). Besides, the mRNA expression level of *DTWD2* was retrieved from TCGA datasets [17], and compared by Pearson's Chi-square test to evaluate its clinical relevance.

### 2.3. Protein Expression Test by Immunohistochemistry Staining

Immunohistochemistry (IHC) staining was performed to evaluate the protein expression level of DTWD2 in COAD tissues. Formalin-fixed, paraffin-embedded specimens were cut into 4 *μ*m slides, deparaffinized, and hydrated. Then, slides were subjected to epitope retrieval, followed by peroxidase incubation to block endogenous reactions. Specific anti-DTWD2 primary antibody (#PA5-62751, Thermo Fisher Scientific; 1 : 100 dilution) was used to incubate with specimen slides overnight at 4°C. Slides were then incubated with horseradish peroxidase (HRP)-linked secondary antibody and subjected to diaminobenzidine staining, followed by counterstaining using Hematoxylin. IHC results were independently assessed by two pathologists to distinguish the high-DTWD2 expression or low-DTWD2 expression of each specimen.

### 2.4. COAD Cell Culture

Two human COAD-originated cell lines (SW480 and SW620) were purchased from ATCC. Cells were cultured in Dulbecco's Modified Eagle Medium (DMEM) medium supplied with 10% fetal bovine serum (FBS) and 1% penicillin/streptomycin [18]. All cells were cultured at 37°C in a humidified atmosphere containing 5% CO_2_. Cells were transfected with pcDNA3.1-DTWD2 plasmids using pcDNA3.1-vector as a negative control by FuGene6 reagent.

### 2.5. Protein Expression Test by Western Blotting

Total protein in cultured cells was extracted with radioimmunoprecipitation assay (RIPA) buffer supplemented with Phenylmethylsulfonyl fluoride (PMSF) and separated by Sodium Dodecyl Sulphate-Polyacrylamide Gel Electrophoresis (SDS-PAGE) and followed by transfer to PolyVinylidene DiFluoride (PVDF) membranes. The membranes were blocked with 5% FBS for 30 minutes at room temperature and incubated with the primary antibodies (anti-DTWD2, #PA5-65902, Thermo Fisher Scientific; anti-glyceraldehyde-3-phosphate dehydrogenase (GAPDH), #2118, Cell Signaling Technology; both 1 : 1000 dilution) at 4°C overnight. Afterward, the membranes were washed and then incubated with HRP-labeled anti-rabbit secondary antibody (#7074, Cell Signaling Technology. 1 : 10,000) for an additional 1 hour at room temperature. Membranes were finally developed using chemiluminescent HRP substrate [19].

### 2.6. 3-(4,5-Dimethylthiazol-2-yl)-2,5-Diphenyl Tetrazolium Bromide Assay

The proliferation assay was conducted as previously described using the 3-(4,5-dimethylthiazol-2-yl)-2,5-diphenyl tetrazolium bromide (MTT) method [20]. Cells were seeded into 96 well plates (5,000 cells/well) and cultured in the regular incubator for designated time points (8, 24, 48, 72, and 96 hours). At each time point, triplicate wells were added with 150 *μ*L of MTT solvent and incubated for another 15 minutes, followed by shaking and pipetting to fully dissolve the MTT formazan. The absorbance value was tested by OD 570 nm.

### 2.7. Proliferation Test by Colony Formation

COAD cells were cultured with a complete medium and seeded in 6-well plates (800 cells/well). Cells were cultured in routine culturing conditions for 14 days to allow colony growth. Afterward, cells were stained with 0.1% crystal violet for 30 minutes and washed [21]. Clones with diameters larger than 1 mm were counted and compared.

### 2.8. Mice Model

BALB/c nude mice were obtained from the Shanghai Animal Center (Shanghai, China). The xenograft model was generated by subcutaneously injecting transfected COAD cells (*n* = 7) or control cells (*n* = 7) into the nude mice [22]. The tumor size was monitored every three days. After three weeks, mice were sacrificed by intraperitoneal injections of 150 mg/kg sodium pentobarbital, and subcutaneous mice xenografts were resected.

### 2.9. Statistics

Cancer-specific survival (CSS) was defined as the time period from disease diagnosis to the date of COAD-related death or the date of last follow-up. Prognostic evaluation was performed using univariate log-rank analysis and multivariate Cox hazard regression analysis, respectively. The SPSS 20.0 and the GraphPad Prism 5.0 software were utilized for data analyses. *P* < 0.05 was defined as statistical significance. ∗ indicates *P* < 0.05, ∗∗ indicates *P* < 0.01, and ∗∗∗ indicates *P* < 0.001.

## 3. Results

### 3.1. Patients' Characteristics

Here, we retrospectively enrolled 176 COAD cases pathologically diagnosed as TNM stage III (Table 1). The median age was 65 years at the time of disease diagnosis. Among them, 87 cases were older than 65 years old, whereas the other 89 cases were younger than or equal to 65 years old. The case numbers of females (*n* = 86) and males (*n* = 90) were similar. As for the tumor sidedness, 49 cases showed ascending colon localization, 37 cases showed transverse colon localization, 17 cases showed descending colon localization and the other 73 cases showed sigmoid colon localization. The median tumor size was 4.0 cm according to the largest lesion diameter. Based on the pathological results, 128 cases were identified as differentiation grade I–II and the other 48 cases as differentiation grade III–IV. There are 27, 113, and 36 cases diagnosed with T stage T1–T2, T3, and T4, respectively. All cases showed positive lymph node metastasis, including 127 N1 stage patients and 49 N2 stage patients. None of the patients accepted pre-operative chemotherapy or radiotherapy. In other words, all 176 enrolled patients were treated with surgical resection first. After that, 73 patients accepted chemotherapy while the other 103 patients did not accept chemotherapy (*n* = 87) or were unsure (*n* = 16). Among the 73 cases who accepted chemotherapy, 14 were treated with FOLFIRI, 21 were treated with XELOX, and 35 were treated with FOLFOX, whereas the other 3 cases were with unknown chemotherapy regimens.

### 3.2. Protein Expression and Clinical Relevance of DTWD2 in COAD

**Figure 1:**
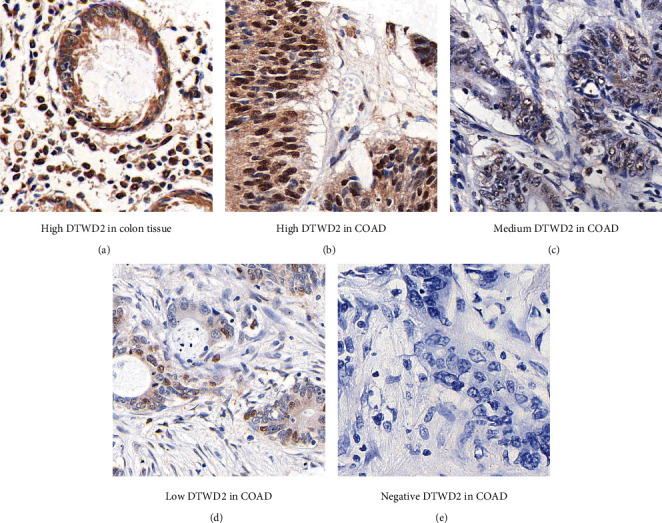
Immunoreaction of DTWD2 in tissue specimens. (a) Representative high protein expression of DTWD2 in adjacent nontumorous colon tissue. (b) Representative high protein expression of DTWD2 in COAD specimen. (c) Representative medium expression of DTWD2 in COAD. (d) Representative low expression of DTWD2 in COAD. (e) Representative negative expression of DTWD2 in COAD. Magnification: 400×.

As described in the method section, COAD samples and adjacent non-tumorous samples were subjected to IHC experiments to test the protein expression level of DTWD2 (Figures 1(a), 1(b), 1(c), 1(d), and 1(e)). According to the diverse immunoreactivity, we sub-classified patients into low-DTWD2 subgroup (*n* = 83) and high-DTWD2 subgroup (*n* = 93). Statistical analysis identified no significant correlation between DTWD2 protein expression and patients' age, sex, tumor localization, tumor pathological grade, lymph node metastasis, and chemotherapy (*P* > 0.05; Table 2). Nevertheless, low DTWD2 expression showed significant crosstalk with larger tumor size (*P* = 0.002), implying that DTWD2 may participate in tumor growth. Consistently, patients with more advanced invasion depth, namely the T stage, showed lower DTWD2 expression levels (*P* < 0.001).

### 3.3. Survival Analysis

**Figure 2:**
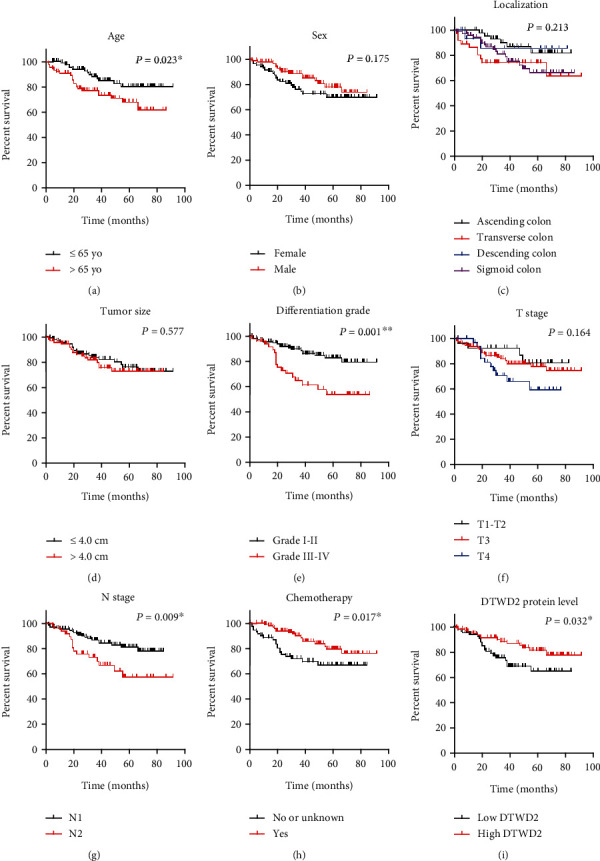
CSS analysis of COAD cohort. The CSS of our enrolled COAD cohort (*n* = 176) was assessed by Kaplan–Meier method according to different subgroups including age (a), sex (b), lesion localization (c), tumor size (d), tumor differentiation grade (e), T stage (f), N stage (g), chemotherapy (h), and DTWD2 protein expression level (i).

Prognoses of the enrolled COAD cohort were assessed by CSS using univariate analysis (Table 3) and multivariate analysis (Table 4), respectively. As indicated by Kaplan–Meier univariate analysis, patients' survival was negatively correlated with their age at the time of diagnosis (*P* = 0.023). The CSS time of younger patients was 79.5 ± 2.9 months with a 5-year CSS rate of 80.5%, whereas was only 64.9 ± 3.7 months with a 5-year CSS rate of 68.5% (Figure 2(a); Table 3). In contrast with patients' age, our cohort did not find any significant survival difference between females and males (Figure 2(b); *P* = 0.175). Although previous studies suggested an indispensable effect of tumor sidedness on COAD prognosis, our cohort did not identify any significant difference among the subgroups (Figure 2(c); *P* = 0.213). The 5-year CSS rates of patients with larger tumor size or smaller tumor size were similar (Figure 2(d), 73.2% *vs.* 76.5%, *P* = 0.577), despite the former ones exhibiting a 6.6-month shorter CSS time (68.0 ± 3.3 months *vs.*75.4 ± 3.3 months). As expected, patients with poor-differentiation grade (grade III) or undifferentiation grade (grade IV) exhibited significantly worse prognosis than those with well-differentiation (grade I) or moderate-differentiation (grade II). The 5-year CSS rate of grade I–II patients was 82.6%, whereas declined to only 54.3% of grade III–IV patients (Figure 2(e), *P* = 0.001). The median CSS time of patients with T stage T1–T2, T3, and T4 were 72.4 ± 4.5, 75.4 ± 3.0, and 57.0 ± 4.6 months; resulting in 5-year CSS rates as 80.9%, 78.0%, and 59.6%, respectively (Figure 2(f), *P* = 0.164). Since all cases enrolled were TNM stage III patients with positive lymph node metastasis, we compared the survival difference between patients with the N1 stage and the N2 stage (Figure 2(g), *P* = 0.009). In detail, patients with the N1 stage showed a 5-year CSS time of 72.4 ± 2.4 months and a 5-year CSS rate of 81.0%, whereas patients with the N2 stage showed a 5-year CSS time of 64.1 ± 5.3 months and a 5-year CSS rate of 57.5%. Our data also confirmed that chemotherapy treatment was beneficial for patients' CSS (Figure 2(h), *P* = 0.017). In the subgroup that accepted chemotherapy, patients' 5-year CSS time was 78.6 ± 2.8 months with a 5-year CSS rate of 79.6%; whereas in the subgroup without chemotherapy or unsure about the treatment, patients' 5-year CSS time was only 62.7 ± 4.0 months with 5-year CSS rate of 67.0%. Importantly, here we identified the significant effect of DTWD2 protein expression in COAD prognosis for the first time (Figure 2(i), *P* = 0.032). Patients with high-DTWD2 protein level showed a median 5-year CSS time of 78.5 ± 2.9 months and a 5-year CSS rate of 79.6%, whereas those with low-DTWD2 level showed a median 5-year CSS time of 63.8 ± 3.7 months and a 5-year CSS rate 65.4%.

We next conducted a multivariate analysis to map independent risk factors for COAD prognosis. As shown in Table 4, poor differentiation grade and lower DTWD2 protein level were identified as two independent unfavorable risk factors for CSS of COAD. The hazard ratio of grade III–IV versus grade I–II was 2.25 (95% CI 1.11–4.57, *P* = 0.024). The hazard ratio of high DTWD2 versus low DTWD2 expression was 0.50 (95% CI 0.25–0.97, *P* = 0.040), indicating that DTWD2 may be a tumor-suppressive factor for COAD.

### 3.4. Clinical Relevance and Prognostic Role of *DTWD2*-mRNA

**Figure 3:**
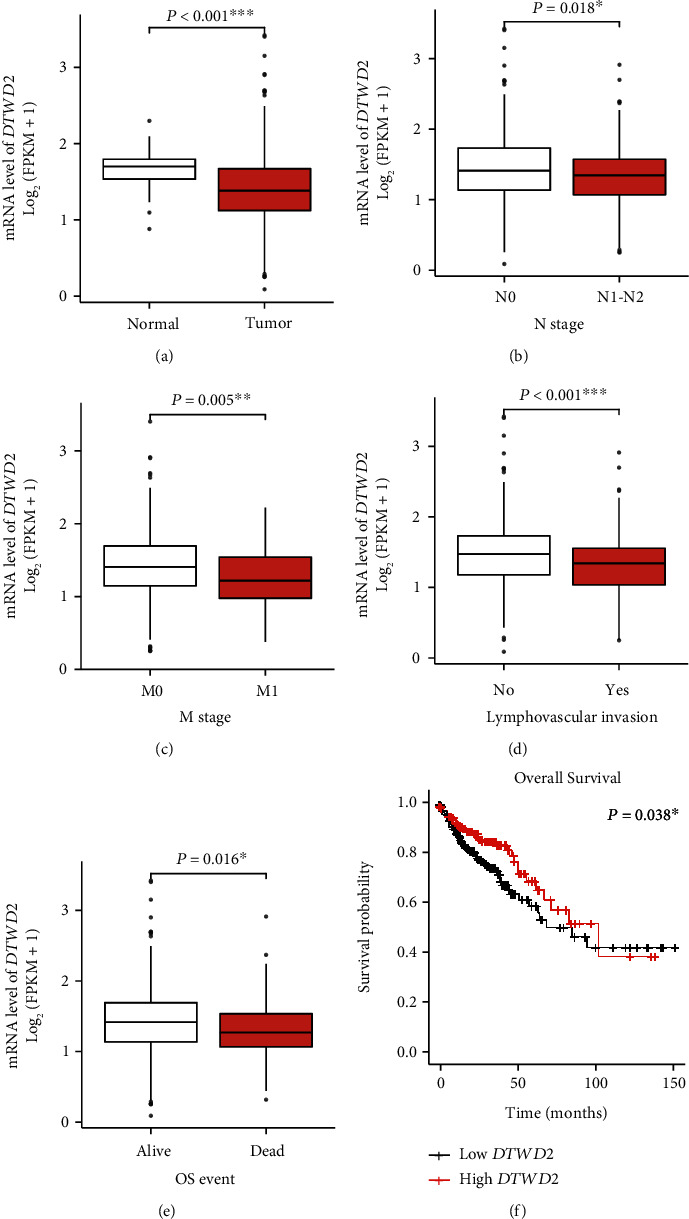
Clinical relevance of *DTWD2*-mRNA in TCGA-COAD cohort. The DTWD2-mRNA level in COAD cohort from TCGA dataset was retrieved and compared based on disease status (a, normal tissue *vs.* tumor tissue), pathological N stage (b, N0 *vs*. N1–N2), clinical M stage (c, M0 *vs.* M1), lymphovascular invasion (d, negative *vs.* positive), patients' survival status (e, alive *vs.* dead). In addition, prognostic role of *DTWD2*-mRNA level on the overall survival of TCGA-COAD cohort was evaluated using Kaplan–Meier method (f).

Considering that our data was collected from a single medical center and only contained COAD cases with TNM stage III, we further investigated the role of DTWD2 in the TCGA cohort, which contains 41 normal colon specimens and 478 COAD specimens. *In silico* analysis firstly suggested that *DTWD2*-mRNA level was lower in COAD tissues than in normal colon tissues (Figure 3(a), *P* < 0.001). Second, *DTWD2*-mRNA was negatively correlated with N stage, M stage, lymphovascular invasion, and disease mortality (Figures 3(b), 3(c), 3(d), and 3(e), all *P* < 0.05). Thirdly, Kaplan–Meier survival analysis indicated that low *DTWD2*-mRNA level was significantly correlated with worse overall survival of COAD (Figure 3(f), *P* = 0.038), which was consistent with our findings regarding its protein expression level.

### 3.5. DTWD2 Inhibits COAD Cell Proliferation

**Figure 4:**
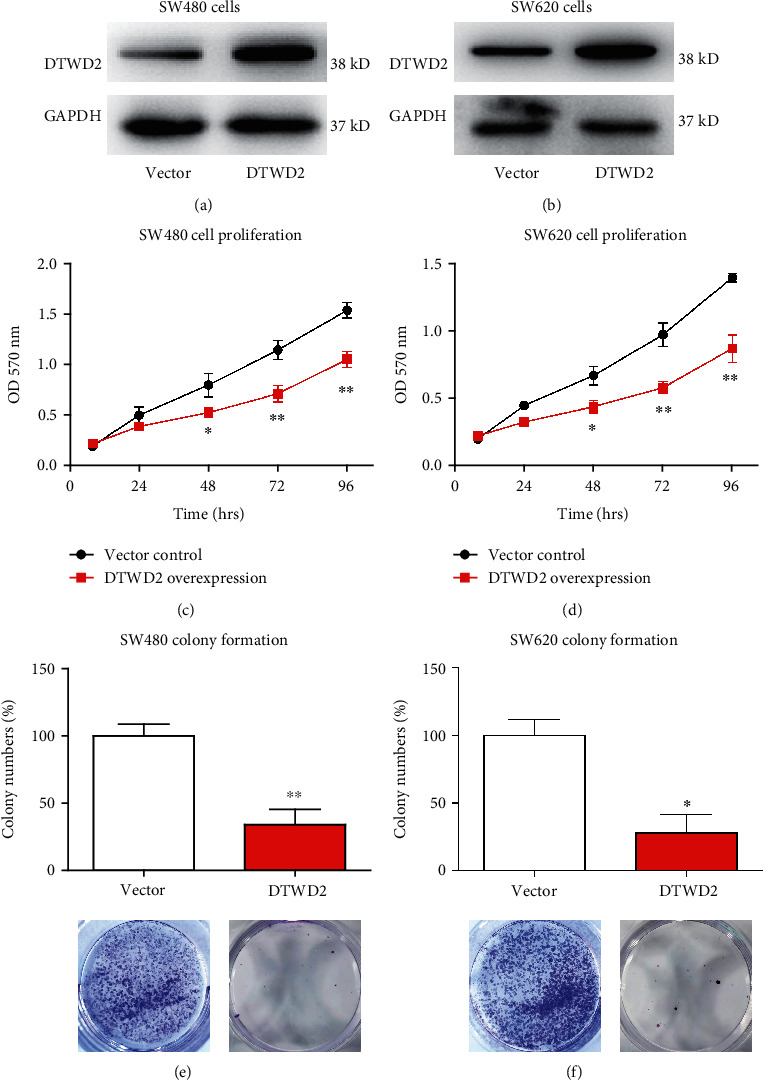
DTWD2 exerts tumor-suppressing effects in COAD cells. (a, b) Overexpression of DTWD2 was achieved by transfecting its coding gene into SW480 and SW620 cell lines, using scrambled vector as control. The transfection efficiency was examined by WB. (c, d) To evaluate proliferation alterations of cells transfected with DTWD2 or vector, MTT method was conducted to plot their proliferation curves. (e, f) Colony formation experiments were conducted to compare colony formation capacities of COAD cells transfected with DTWD2 or vector.

Based on the clinical data, we hypothesized that DTWD2 may exert tumor-suppressing effects in COAD. Therefore, we next conducted cellular experiments by ectopic expression of DTWD2. Plasmids containing the coding gene of DTWD2 were transfected into SW480 and SW620 cells, using scrambled vector plasmids as controls. After confirming the overexpressing efficiency of plasmids by western blotting (WB) (Figures 4(a) and 4(b)), cells were further subjected to phenotype analyses. For example, cell proliferation capacity was evaluated by MTT experiments, which demonstrated that overexpressing DTWD2 resulted in decreased cell proliferation (Figures 4(c) and 4(d)). Consistently, colony formation assay indicated that overexpressing DTWD2 could significantly attenuate the colony formation capacity of both SW480 and SW620 cells (Figures 4(e) and 4(f)).

### 3.6. DTWD2 Suppresses COAD Growth in Mice Model

**Figure 5:**
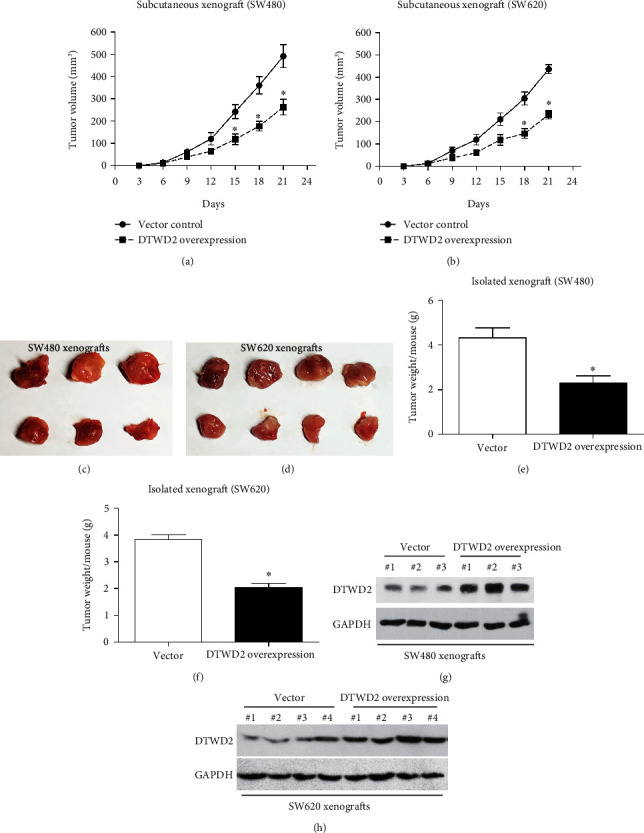
DTWD2 attenuates COAD growth in mice model. (a, b) Cells transfected with DTWD2 plasmids or vector plasmids were subcutaneously injected into nude mice to establish xenograft mice model. Then the growth curves were plotted every three days. (c, d) The images showed size difference of resected xenografts. (e, f) The weights of resected xenografts were tested and compared by unpaired Student's *t*-test. (g, h) WB was conducted to double-confirm DTWD2's expression difference in resected xenografts.

Furthermore, we established a mice xenograft model in nude mice to validate *the in vivo* effects of DTWD2 during COAD progression. COAD cells with ectopic high expression of DTWD2 or control cells were subcutaneously injected into nude mice to monitor xenograft growth, which showed that DTWD2-overexpression inhibits COAD growth in mice model (Figures 5(a) and 5(b)). Three weeks later, all xenografts were resected and exhibited consistent size differences with growth curves above (Figures 5(c) and 5(d)). In addition, we tested the weights of all resected tumors and came to the same conclusion (Figures 5(e) and 5(f)). Finally, we lysed the resected tumors and tested protein expression level of DTWD2 to validate the overexpression efficiencies. As expected, xenografts generated by DTWD2-overexpressing cells showed significantly higher DTWD2 immunoreactivities than the control ones (Figures 5(g) and 5(h)).

## 4. Discussion

There are hundreds of tRNA modifications in mammals, which are similar to several well-known protein modifications, such as phosphorylation and ubiquitination [23]. DTWD protein family refers to a specific protein family that can catalyze the formation of acp^3^U of tRNAs, an important post-translational modification of tRNA. Abnormal regulation of tRNA modifications leads to impaired cell growth [24]. Previous studies suggested tumor-related roles of DTWDs in different malignancies. For example, DTWD1 has been discovered as a tumor suppressor, whose inactivating mutations had been identified in colorectal cancer [25]. Here, we initially discovered the significant role of DTWD2 in human cancer. According to the TCGA dataset and another COAD cohort from our medical center, we confirmed that DTWD2 was significantly downregulated in COAD specimens compared to non-tumorous colon tissues. Meanwhile, low mRNA levels or protein level of DTWD2 can help predict a worse prognosis of COAD cases.

Besides the novel finding regarding the prognostic predictive role of DTWD2 in COAD, our cohort revealed several interesting points in TNM stage III COADs. Firstly, patients' age affected the CSS of COAD. This is unexpected since most published studies reported the correlation between patients' age and OS instead of CSS. In our opinion, this finding is also reasonable because older age leads to decreased cancer immunity, subsequently resulting in a higher risk of disease relapse or heterochronous metastasis.

Moreover, our study provided the initial evidence that overexpressing DTWD2 could significantly attenuate COAD cell proliferation and colony formation capacities. The in vitro experimental data was further validated by in vivo mice experiments, highlighting that targeting DTWD2 may be a novel direction for COAD treatment, especially for those with dysregulated DTWD2 expression.

Our study has several limitations. First, the retrospective cohort from our hospital only contained TNM stage III COAD cases, thus our conclusion may only apply to COADs with locally advanced stages. However, we retrieved the *DTWD2*-mRNA information in the TCGA cohort which contains COAD cases in TNM stage I–IV. Of note, the major conclusion that lower DTWD2 predicted a worse prognosis was consistent in our stage III-COAD cohort and COAD-TCGA cohort. Therefore, the tumor-related role of DTWD2 in more malignancy types deserves further investigation. Second, despite our data confirming the tumor-suppressing effects of DTWD2 in COAD cells and mice models, its detailed signaling mechanisms remain unclear. Considering that transfected cancer cells exhibited a lower proliferation rate and a reduced colony formation capacity compared to the control group. We further examined several signaling pathways known to regulate cell proliferation, such as the MAPK/ERK and PI3K/Akt pathways, as well as cell cycle-related proteins, including cyclins and CDKs [26]. Unfortunately, we did not observe any significant alteration of the abovementioned pathways (data not shown). To further dig into the potential downstream signaling mechanisms, high throughput screening experiments, such as RNA-sequencing and mass-spectrometry, will be necessary. In our hypothesis, as a tRNA-uridine aminocarboxypropyltransferase, DTWD2 may participate in tRNA post-transcriptional modification and play multiple functions in various aspects. WB may be incapable of detecting slight changes due to its low resolution. Furthermore, multiple-omic strategies will be essential to map and elucidate the detailed signaling network of DTWD2 in tumor cells.

## 5. Conclusions

DTWD2 inhibits COAD progression by suppressing tumor growth both *in vitro* and *in vivo*. Low expression of DTWD2 in tumor tissues predicts worse clinical outcomes of COAD.

## Figures and Tables

**Table 1:** Patients' information.

Variables	Cases (*n* = 176)	Percentage (%)
Age (years)		
≤65	89	50.6
>65	87	49.4
Sex		
Female	86	48.9
Male	90	51.1
Tumor location		
Ascending colon	49	27.8
Transverse colon	37	21.0
Descending colon	17	9.7
Sigmoid colon	73	41.5
Tumor size (cm)		
≤4.0	88	50.0
>4.0	88	50.0
Differentiation grade		
Grade I–II	128	72.7
Grade III–IV	48	27.3
T stage		
T1–T2	27	15.3
T3	113	64.2
T4	36	20.5
N stage		
N1	127	72.2
N2	49	27.8
Chemotherapy		
Not accepted chemotherapy	87	49.4
Unsure accepted or not	16	9.1
FOLFIRI-chemotherapy	14	8.0
XELOX-chemotherapy	21	11.9
FOLFOX-chemotherapy	35	19.9
Unknown chemotherapy regimen	3	1.7

**Table 2:** Correlations between characteristics of TNM Stage III COAD patients and DTWD2 protein expression.

Variables	Cases	DTWD2 protein expression		*P* value
	(*n* = 176)	Low (*n* = 83)	High (*n* = 93)	
Age (years)				
≤65	89	39	50	0.369
>65	87	44	43	
Sex				
Female	86	39	47	0.638
Male	90	44	46	
Tumor location				
Ascending colon	49	23	26	0.940
Transverse colon	37	17	20	
Descending colon	17	7	10	
Sigmoid colon	73	36	37	
Tumor size (cm)				
≤4.0	88	31	57	0.002∗∗
>4.0	88	52	36	
Differentiation grade				
Grade I–II	128	62	66	0.579
Grade III–IV	48	21	27	
T stage				
T1–T2	27	2	25	<0.001∗∗∗
T3	113	61	52	
T4	36	20	16	
N stage				
N1	127	59	68	0.764
N2	49	24	25	
Chemotherapy				
No or unknown	103	43	60	0.088
Accepted	73	40	33	

^∗∗^ *P* < 0.01, and ^∗∗∗^*P* < 0.001.

**Table 3:** CSS analyses of enrolled TNM Stage III COAD patients.

Variables	Cases (*n* = 176)	Survival months (mean ± S.D.)	5-year CSS (%)	*P* value
Age (years)				
≤65	89	79.5 ± 2.9	80.5	0.023∗
>65	87	64.9 ± 3.7	68.5	
Sex				
Female	86	70.8 ± 3.8	70.4	0.175
Male	90	71.9 ± 2.8	78.3	
Tumor location				
Ascending colon	49	75.3 ± 3.3	82.1	0.213
Transverse colon	37	68.1 ± 6.1	74.7	
Descending colon	17	71.3 ± 6.4	85.6	
Sigmoid colon	73	67.5 ± 3.7	66.5	
Tumor size (cm)				
≤4.0 cm	88	75.4 ± 3.3	76.5	0.577
>4.0 cm	88	68.0 ± 3.3	73.2	
Differentiation grade				
Grade I–II	128	79.0 ± 2.6	82.6	0.001∗∗
Grade III–IV	48	58.5 ± 5.0	54.3	
T stage				
T1–T2	27	72.4 ± 4.5	80.9	0.164
T3	113	75.4 ± 3.0	78.0	
T4	36	57.0 ± 4.6	59.6	
N stage				
N1	127	72.4 ± 2.4	81.0	0.009∗
N2	49	64.1 ± 5.3	57.5	
Chemotherapy				
No or unknown	103	78.6 ± 2.8	79.6	0.017∗
Accepted	73	62.7 ± 4.0	67.0	
DTWD2 protein level				
Low	83	63.8 ± 3.7	65.4	0.032∗
High	93	78.5 ± 2.9	81.7	

^∗∗^ *P* < 0.01, and ^∗∗∗^*P* < 0.001.

**Table 4:** CSS analyses of enrolled TNM Stage III COAD patients.

Variables	Cases (*n* = 176)	Survival months (mean ± S.D.)	5-year CSS (%)	*P* value
Age (years)				
≤65	89	79.5 ± 2.9	80.5	0.023∗
>65	87	64.9 ± 3.7	68.5	
Sex				
Female	86	70.8 ± 3.8	70.4	0.175
Male	90	71.9 ± 2.8	78.3	
Tumor location				
Ascending colon	49	75.3 ± 3.3	82.1	0.213
Transverse colon	37	68.1 ± 6.1	74.7	
Descending colon	17	71.3 ± 6.4	85.6	
Sigmoid colon	73	67.5 ± 3.7	66.5	
Tumor size (cm)				
≤4.0 cm	88	75.4 ± 3.3	76.5	0.577
>4.0 cm	88	68.0 ± 3.3	73.2	
Differentiation grade				
Grade I–II	128	79.0 ± 2.6	82.6	0.001∗∗
Grade III–IV	48	58.5 ± 5.0	54.3	
T stage				
T1–T2	27	72.4 ± 4.5	80.9	0.164
T3	113	75.4 ± 3.0	78.0	
T4	36	57.0 ± 4.6	59.6	
N stage				
N1	127	72.4 ± 2.4	81.0	0.009∗
N2	49	64.1 ± 5.3	57.5	
Chemotherapy				
No or unknown	103	78.6 ± 2.8	79.6	0.017∗
Accepted	73	62.7 ± 4.0	67.0	
DTWD2 protein level				
Low	83	63.8 ± 3.7	65.4	0.032∗
High	93	78.5 ± 2.9	81.7	

^∗∗^ *P* < 0.01, and ^∗∗∗^*P* < 0.001.

## Data Availability

Data supporting this research article are available from the corresponding author or first author on reasonable request.
